# Transactions of the Fifty-first Session of the American Institute of Homœpathy

**Published:** 1896-03

**Authors:** 


### BOOK NOTICES.

TRANSACTIONS OF THE FIFTY-FIRST SESSION OF THE AMERICAN INSTITUTE OF HOMŒOPATHY. Held at Newport, R. I., June 20th to 26th, 1895. Edited by Eugene H. Porter, M. A., M. D., General Secretary, 181 West Seventy-third Street, New York City. Sherman & Co., Printers, Seventh and Cherry Streets, Philadelphia, 1895.

This is an immense volume of twelve hundred pages. It contains an accurate report of the proceedings of the Institute, together with all the papers presented. The proceedings of the different sections are given in detail, both the contributions and the interesting discussions. It is a moral impossibility to give any extended criticism of these articles or the discussions upon them. We note, however, some excellent illustrations. Several of these are photographs, intended to amplify an excellent article on Astigmatism, by Dr. Harold Wilson. Others are wood engravings, for an article upon the Surgery of the Intestinal Tract, by Dr. Horace Packard. The work of the Institute is so great at each meeting that it was found necessary to divide it into bureaus, as is perhaps well known. The articles, therefore, are grouped under their appropriate bureaus, and thus an association is established of those papers that relate to kindred subjects. The material contained in these bureaus is certainly very interesting and instructive.

